# Comparative 3D Genome Structure Analysis of the Fission and the Budding Yeast

**DOI:** 10.1371/journal.pone.0119672

**Published:** 2015-03-23

**Authors:** Ke Gong, Harianto Tjong, Xianghong Jasmine Zhou, Frank Alber

**Affiliations:** Molecular and Computational Biology, Department of Biological Sciences, University of Southern California, 1050 Childs Way, Los Angeles, CA 90089, United States of America; Duke University, UNITED STATES

## Abstract

We studied the 3D structural organization of the fission yeast genome, which emerges from the tethering of heterochromatic regions in otherwise randomly configured chromosomes represented as flexible polymer chains in an nuclear environment. This model is sufficient to explain in a statistical manner many experimentally determined distinctive features of the fission yeast genome, including chromatin interaction patterns from Hi-C experiments and the co-locations of functionally related and co-expressed genes, such as genes expressed by Pol-III. Our findings demonstrate that some previously described structure-function correlations can be explained as a consequence of random chromatin collisions driven by a few geometric constraints (mainly due to centromere-SPB and telomere-NE tethering) combined with the specific gene locations in the chromosome sequence. We also performed a comparative analysis between the fission and budding yeast genome structures, for which we previously detected a similar organizing principle. However, due to the different chromosome sizes and numbers, substantial differences are observed in the 3D structural genome organization between the two species, most notably in the nuclear locations of orthologous genes, and the extent of nuclear territories for genes and chromosomes. However, despite those differences, remarkably, functional similarities are maintained, which is evident when comparing spatial clustering of functionally related genes in both yeasts. Functionally related genes show a similar spatial clustering behavior in both yeasts, even though their nuclear locations are largely different between the yeast species.

## Introduction

The 3D structural organization of the genome plays a key role in the correct execution of nuclear functions, such as gene expression regulation[1–4], and DNA replication[5,6]. In yeast, centromeres remain attached to the spindle pole body (SPB) during interphase, and telomeres are typically anchored to nuclear envelope (NE)[7–11]. For budding yeast, chromosomes were described of having a “Rabl-like” orientation and genes are located in defined nuclear territories[12–15]. Moreover, the rDNA containing nucleolus is located in a well-defined position relative to the SPB[16–18]. More recently, conformation capture experiments (Hi-C methods) [19–21] provided a detailed view of the genome-wide chromatin interaction patterns in budding yeast (*Saccharomyces cerevisiae*) and fission yeast (*Schizosaccharomyces pombe*). The Hi-C experiments revealed large differences between the two yeast types [22–25]. Budding yeast showed highly structured contact maps with distinct cross-shaped patterns for intra-and inter-chromosomal interactions. In comparison, the Hi-C maps of fission yeast show only weakly structured patterns, and, with the exception of centromere and telomere interactions, are dominated by local chromosome chain contacts. Several studies correlated Hi-C interaction patterns with functional features [22,23,26–28]. For instance, some co-regulated genes in fission yeast form frequent interactions, and are assumed to be spatially clustered even though they are substantially separated in the genome sequence [23].

We and others recently discovered that in budding yeast, entirely random configurations of tethered chromosomes are sufficient to reproduce in a statistical manner many data about the budding yeast genome organization[29–33], including gene loci interactions from genome-wide Hi-C experiments [22], the distribution of gene territories from fluorescence imaging [12],and the clustering of functionally related genes such as replication start sites as well as tRNA genes [22,34,35]

Here, we focus on fission yeast (*Schizosaccharomyces pombe*) and investigate the role of geometric constraints on its genome organization for the given gene order on each chromosome. To fairly assess the factors responsible for genome structure-function correlations, we must first examine the genome structure that arises when chromosomes are tethered to nuclear landmarks but otherwise randomly configured in the confinement of the nuclear environment. Like budding yeast, the fission yeast centromeres are attached to the SPB during interphase through microtubule interactions. The fission yeast telomeres are anchored to the NE and the SPB is located at opposite sides from the nucleolus, which contains rDNA genes [11,18]. All these factors exert geometric constraints on the chromosome fibers. Although the genomes of budding and fission yeast are almost of equal size (~12Mb), the total number and length of the chromosomes are largely different. Fission yeast has only 3 chromosomes compared to the 16 in budding yeast and they are significantly larger [36]. Due to these changes, the impact of geometric constraints on the chromosome conformations is different between the two yeasts, and it is unknown if random encounters of constraint chromosomes alone could explain the observed fission yeast genome structure and structure-function correlations.

Here, we calculated a large population of fission yeast genome structures in which chromosomes are constrained by geometric constraints but otherwise randomly configured in the nucleus. We quantitatively characterized the resulting chromatin contact patterns, nuclear territories of gene loci and chromosomes, and also analyzed structure function correlations including the co-locations of co-expressed and functionally related genes. Our findings demonstrate that purely random configurations of flexible chromosome chains, combined with the locations of genes on the chromosomes can reproduce a wide range of experimental data, including chromatin interaction patterns and locations from Hi-C and FISH experiments, such as the spatial clustering of tRNA and 5sRNA genes, as well as clustering of co-expressed genes.

Although fission and budding yeast genomes share similar principles of genome organization, a comparative structure analysis revealed dramatic differences in the resulting structure populations. For instance, almost all chromatin regions in fission yeast can access wide areas in the nuclear volume, whereas in budding yeast only a small fraction of loci show a similar behavior. Therefore, in this model gene territories in fission yeast are generally more diffused than those in budding yeast and nuclear locations of orthologous genes can be quite different between them. However, despite the structural differences, the clustering behavior of related genes is quite similar in fission and budding yeast, even though the genes’ nuclear locations differ. Moreover, our analysis also provides insights on the contribution of individual constraint types in establishing functional relevant interaction patterns. For instance, centromere clustering is particularly important in establishing inter-chromosomal clustering of tRNA genes in fission yeast. In summary, our findings demonstrate that some described structure-function correlations can be explained as a consequence of random chromatin collisions driven by a few geometric constraints (mainly centromere-SPB and telomere-NE tethering), combined with the specific gene locations in the chromosome sequence.

## Materials and Methods

**Fig 1:**
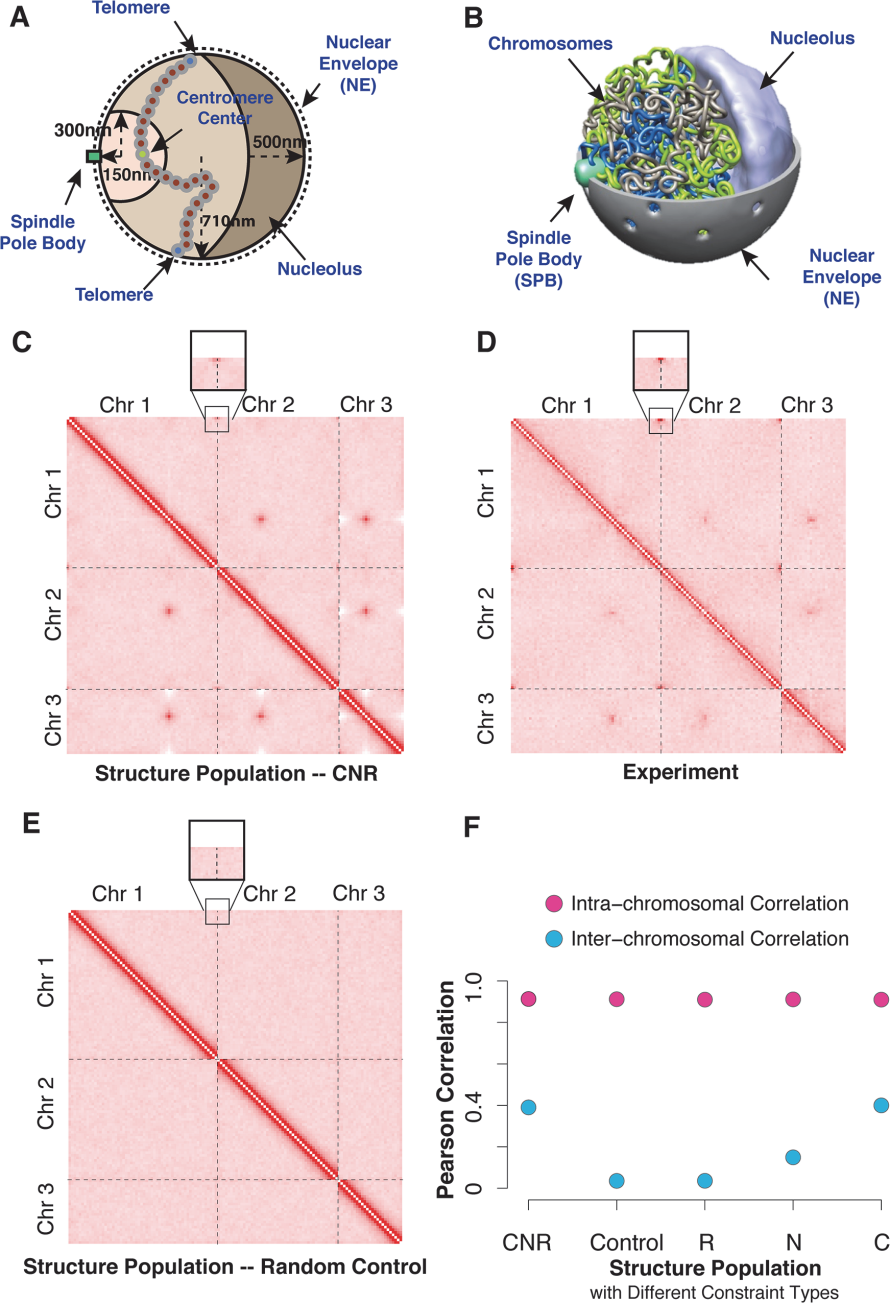
Fission yeast genome structures calculation. **(A)** Schematic view of fission yeast nuclear architecture and imposed geometric constraints. Centromeres are located within a sphere volume of radius 300 nm to ensure that they are close to the SPB. Telomeres are anchored to the NE and can freely move on the NE surface. rDNA genes are constrained to be on the nucleolar surface (right side). All non-rDNA genes are prevented to enter the nucleolus. All chromosomes are confined in a nucleus of radius 0.71 micron. **(B)** Snapshot of a genome structure illustrating the packing of the chromosomes in the nuclear volume. Different chromosome chains are depicted in different colors. The nucleolus volume is shown in silver. The spindle pole body is shown as a light green cylinder opposite to nucleolus. **(C,D,E)** Heatmaps of the genome-wide contact frequencies of the fission yeast from calculated structure populations (**C**), from experiment (**D**), and from a random control model with no geometric constraints applied (**E**). The resolution of the heatmaps is 96 kb per bin (S1 Text). The color code ranges from white to red to represent frequencies from low to high. The telomere-telomere interactions are highlighted in a zoom-in box **(F)** Pearson correlation between contact frequency heat maps from experiment and structure populations (S1 Text). Correlation values are shown for intra-chromosomal and inter-chromosomal interactions separately. The experimental heat map is compared to several different structure populations generated with different amount of geometric constraints. Values for models C, R, N indicate structure populations that were generated only with centromeric constraints (C), rDNA constraints (R) and telomere anchoring constraints (N), respectively (Methods).

In our model, the yeast nuclear architecture is defined by the nuclear envelope (NE), the spindle pole body (SPB), the nucleolus, and the 3 chromosomes for the haploid fission yeast genome (Fig. 1A). The positions of the NE, SPB and nucleolus remain constant while the configurations of the chromosomes are optimized.

### Chromosome Representation

Each chromosome is represented by a flexible chromatin fiber as a chain of connected spheres as described previously [29]. Each bead with radius of 15 nm represents 3.2 kb of genome sequence, which corresponds to a fiber compaction ratio of 6 nucleosomes per 11 nm fiber length [29,37]. The entire genome contains 3930 beads.

### Nuclear Architecture

The nuclear radius (*r* = 710 nm) and the position of the SBP and nucleolus are based on data from imaging experiments[11,23]. The SPB and nucleolus are positioned at opposite ends of the nucleus (Fig. 1B).

### Scoring Function

The geometrical constraints are expressed in a single scoring function, which is defined as a sum of spatial restraints and quantifies the degree of consistency between the structure and the constraints. To optimize an individual structure, the scoring function is minimized to a score of ~zero therefore entirely satisfying the restraints by a small residual. The scoring function is defined as

$$\begin{array}{l} S(r_{i},..,r_{N})=\sum_{i=1,i\notin \alpha }^{N-1}U_{\text{ch}}(r_{i},r_{i+1})+\sum_{i=1}^{N-1}\sum_{j>i}^{N}U_{\text{exc}}(r_{i},r_{j})+\sum_{i=1}^{N}U_{\text{nuc}}(r_{i})\\ \;\;\;\;\;\;+\sum_{i\in \beta }U_{\text{cen}}(r_{i})+\sum_{i\in \gamma }U_{\text{tel}}(r_{i})+\sum_{i\in \delta }U_{\text{inu}}(r_{i})+\sum_{i\notin \delta }U_{\text{onu}}(r_{i})\\ \;\;\;\;\;\;=0 \end{array}.$$

Here, **r**_*i*_ ∊ ℜ^3^ is the 3D coordinate vector of each bead *i; N* is the total number of beads in a model and α, β, γ, and δ represent different subsets of beads with specific genomic features. α is the set of all terminal beads of the chromosomes; β is the set of all centromeric beads; γ is the set of beads representing telomeres, and δ are all beads representing rDNA regions (detailed explanation in Table 1). All the restraints are expressed as pseudo potential energy terms *u* described as follows:

*Chromatin chain restraints U*_ch_ restricts consecutive beads in a chromosome chain to be within a distance of 30nm. (Table 1).

*Chromatin chain excluded volume restraints U*_exc_ prevents the overlap between any two beads in the genome (Table 1).

*Nuclear envelope restraints U*_nuc_ ensures that all beads reside inside the nucleus with a radius of *R*_nuc_ = 710nm (Table 1).

*Chromatin persistence length*. A harmonic potential restraint is imposed to reproduce the desired chain stiffness during the optimization process.

$$U_{\text{angle}}=\frac{1}{2}k_{\text{angle}}\sum_{i=1}^{N-2}\left(1-\arccos(({\text{r}}_{i+1}-{\text{r}}_{i})↺({\text{r}}_{i+2}-{\text{r}}_{i+1}))\right),\;\mathrm{for}\;i,i+1,\text{and}\;i+2\;\text{on the same chain}.$$

**Table 1:** Geometric constraints and their functional forms.

Restraint Type		Functional form	Μ	*d*(nm)	bead *i*	k
Non-Specific Geometric Restraints						
*U*_n_	*Beads inside nucleus restraint*	*u* ^ub^	(0,0,0)	710	all beads	1
*U*_ch_	*Chromatin chain bond restraint*	*u* ^h^	ri+1	30	*i* = {1..*N* – 1}, *i* + 1 *∉ α*	1
*U*_exc_	*Excluded volume restraint*	*u* ^lb^	Rj	30	*i* = {1,..*N* – 1}, *j* > *i*	1
Specific Geometric Restraints						
*U*_cen_	*Centromere localization restraint (C)*	*u* ^ub^	(−560,0,0)	300	*∀i* ∊ *β*	1
*U*_onu_	*rDNA restraint 1outside nucleolus (R)*	*u* ^ub^	(−1065,0,0)	1278	*∀i* ∉ *δ*	1
*U*_inu_	*rDNA restraint 2inside nucleolus (R)*	*u* ^lb^	(−1065,0,0)	1278	*∀i* ∊ *δ*	1
*U*_tel-ne_	*Telomere to NE restraint (N)*	*u* ^lb^	(0,0,0)	710	*∀i* ∊ γ	1

All constraints are expressed as harmonic functions, namely (u^*h*^), harmonic upper (u^*ub*^) and lower bounds (u^*lb*^) [29]. Based on different properties, we define several subsets of beads. α is the set of all end beads for every chromosome chain, *β* is the set of beads representing centromeric regions, *γ* is the set of beads representing telomeric regions, and *δ* is the set of beads which represent the start and end of rDNA regions.

This restraint is only imposed during the calculation of gradient forces in the optimization process and is not considered when calculating the final score (see score function). With a force constant of *k*_angle_ = 0.2 kcal/mol, the obtained chromatin chains have a persistence length as expected for a chromatin fiber, which is assumed to behave similarly as in budding yeast study, for which experimental estimates exist [29].

### Geometric Constraints to Nuclear Landmarks

**(C)** *Centromere localization restraint U*_cen_. All centromeres are clustered at the SPB through interactions with microtubules of length ~300 nm. Therefore centromeres are constrained to be located at the SPB (centromere constraints C) by restricting the central bead of the centromere region to be located in a sphere of radius 300 nm (Fig. 1). Based fluorescence imaging this volume is located on the central axis of the nucleus, close to the NE (Table 1) [11].

**(N)** *Telomere localization restraint U*_tel-NE_. All telomeres are anchored to the NE and can freely move on the NE surface as suggested by experimental evidence (Table 1) [11].

**(R1)** *Nucleolus localization restraint U*_inu_. The ribosomal DNA (rDNA) is located next to the telomeric regions on chromosome 3. Experiments showed that the rDNA regions occupy the nucleolus[11]. We don’t explicitly resolve the structures of rDNA regions in this model. Instead, we anchor the two beads representing the rDNA start and end regions to the surface of nucleolus as previously described for budding yeast[29]. The two anchor points can freely move on the nucleolus surface (Table 1).

**(R2)** *Nucleolus excluded volume restraint U*_onu_. All chromosomal regions other than rDNA repeats are excluded from the nucleolus (Table 1).

To study the influence of each of the geometric constraint types on the genome organization, we generated several structure populations using different combinations of geometric constraints (Table 2, Control, C, N, R, CNR).

**Table 2:** Constraints applied for different models.

*Model Name*	*Centromere Restraints (C)*	*Telomere to NE Restraints (N)*	*rDNA Restraints (R)*
*CNR*	✓	✓	✓
*Control*			
*C*	✓		
*N*		✓	
*R*			✓

In total we generate 5 different models with different combination of specific geometric restraints (Table 1). A check mark indicates that a model contains the corresponding set of constraints.

### Structure optimization

The scoring function is optimized by using a combination of simulated annealing molecular dynamics and the conjugate gradient methods implemented in the Integrated Modeling Platform [29,38,39]. An individual optimization run starts with an entirely random bead configuration, followed by an initial optimization of the structure. Then, we apply simulated annealing protocols to entirely equilibrate the genome configuration. Finally, conjugate gradient optimization ensures that all constraints are satisfied, leading to a structure with score ~zero. Many independent optimizations are carried out to generate a population of at least 100,000 independently calculated genome structures with a total score of ~zero. This population represents a spectrum of genome structures consistent with the input constraints. To test the effect of different constraint types, we generated a total of 4 structure populations with different geometric constraints and 1 structure population without imposing any geometric constraints (random control model) (Table 2).

## Results and Discussion

### Fission yeast genome structure

To represent highly variable genome structures, we constructed a large population of 3D genome structures, which represent a spectrum of all possible chromosome configurations. To explore the chromosome conformational space, 100,000 independent simulations were performed, each time starting with a random genome configuration. After structure optimization and equilibration, each of the independently calculated 100,000 structures satisfies all the imposed geometric constraints. The optimized structures are hereafter referred to as the “structure population” (‘CNR Model’ in Table 2). To investigate the role of the different geometric constraints types, we also generated 3 additional structure populations each containing geometric constraints of only one specific type (‘C Model’, ’N Model’, ’R Model’ in Table 2), and 1 structure population generated without imposing any geometric constraints (‘Random control Model’ in Table 2).

We first discuss the structure population generated with the complete set of geometric constraints (CNR model in Table 2). From the structure population we quantitatively characterized structural features and compared these with experimental data. Specifically we determined the chromosome and loci contact patterns, locus-locus distances, as well as nuclear territories of genes and chromosomes.

### Comparison of contact frequencies to Hi-C experiments

We first compared the contact frequencies calculated from our structure population with those from Hi-C experiments[23,40]. The contact frequency between two chromatin regions is defined as the fraction of all structures containing a contact between the corresponding chain beads (S1 Text). The contact frequencies reproduced well those from the Hi-C experiments, with a Pearson correlation of 0.91 (Fig. 1C, CNR model, Table 2). Also a visual inspection confirms the agreement between model and experiment (Fig. 1C,D).

The intra-chromosomal contact frequency map is dominated by high intensity values along the diagonal, with a sharp decay of frequencies for contact pairs with increasing sequence distance. Long-range intra-chromosomal contacts of a locus appear almost uniformly distributed, similar to an unconstrained polymer. Indeed, the structure population generated without any geometric constraints (‘Control Model’, Table 2) still showed a high Pearson correlation of 0.91 for intra-chromosomal contacts due to the dominant diagonal elements in the contact frequency map (‘Control Model’ in Fig. 1F). The only intra-chromosomal features not recovered in the random control are weak interactions between telomeres (Fig. 1D,E, S1 Fig.). This observation may in part be explained by the relatively large size of the chromosomes. With increasing chromosome size the geometric constraints (i.e., centromere clustering and telomere-NE anchoring) are less restrictive on the conformational flexibility for most of the chromosome regions (i.e., the central regions in a chromosome arm) and therefore have less influence on the intra-chromosomal contact behavior. Therefore regions that are distant in sequence to centromeres or telomeres behave similarly to a random unconstrained polymer. A complete different contact behavior is seen in budding yeast, where the characteristic cross-shaped intra-chromosomal interaction patterns can only be reproduced when geometric constraints are imposed, partly as a result of the smaller chromosome arm lengths [29]. We previously showed that these interaction patterns are mainly a result of crowding effects when centromeres of all 16 chromosomes compete for the limited space around the spindle pole body. Because fission yeast has only three chromosomes a cross-shaped interaction pattern is not observed in model and experiment even though the same type of geometric constraints are imposed.

When analyzing inter-chromosomal interactions a different picture emerges. A random chain model without geometric constraints does not reproduce any of the experimental contact frequencies (Pearson correlation r_P_ ~ 0)(‘Control Model’ in Fig. 1F). Instead, a structure population with geometric constraints reproduced the experimental inter-chromosomal interaction patterns with a Pearson correlation of ~0.40 (‘CNR Model’ in Fig. 1F). The model captures naturally the two key interaction patterns observed in experiment, namely centromere-centromere interactions resulting from centromere clustering, and also an increased telomere-telomere interaction frequency, even though no constraints between telomeres were imposed (Fig. 1C,D, S1 Fig.). Also in budding yeast, significant interactions are observed between telomeres, in particular for the smaller chromosomes [29].

Among all the individual constraint types, centromere clustering leads to the largest increase in correlation between modeled and experimental contact frequency maps (‘C Model’ Fig. 1F). This effect is mainly due to the enhanced contact frequencies between centromeres and a slight decrease in contact frequencies between centromeres and other regions on the chromosome, which is only faintly visible in the experimental heat maps, while it is more pronounced in the modeled structure populations. Other constraint types have only minor impact. For instance, constraining ribosomal genes to the nucleolus does not significantly improve the match between modeled and experimental interactions (Pearson Correlation: 0.03 for ‘R Model’ in Fig. 1D).

We now investigate if other known structural features are also observed in the genome structures.

### Distances between loci

**Fig 2:**
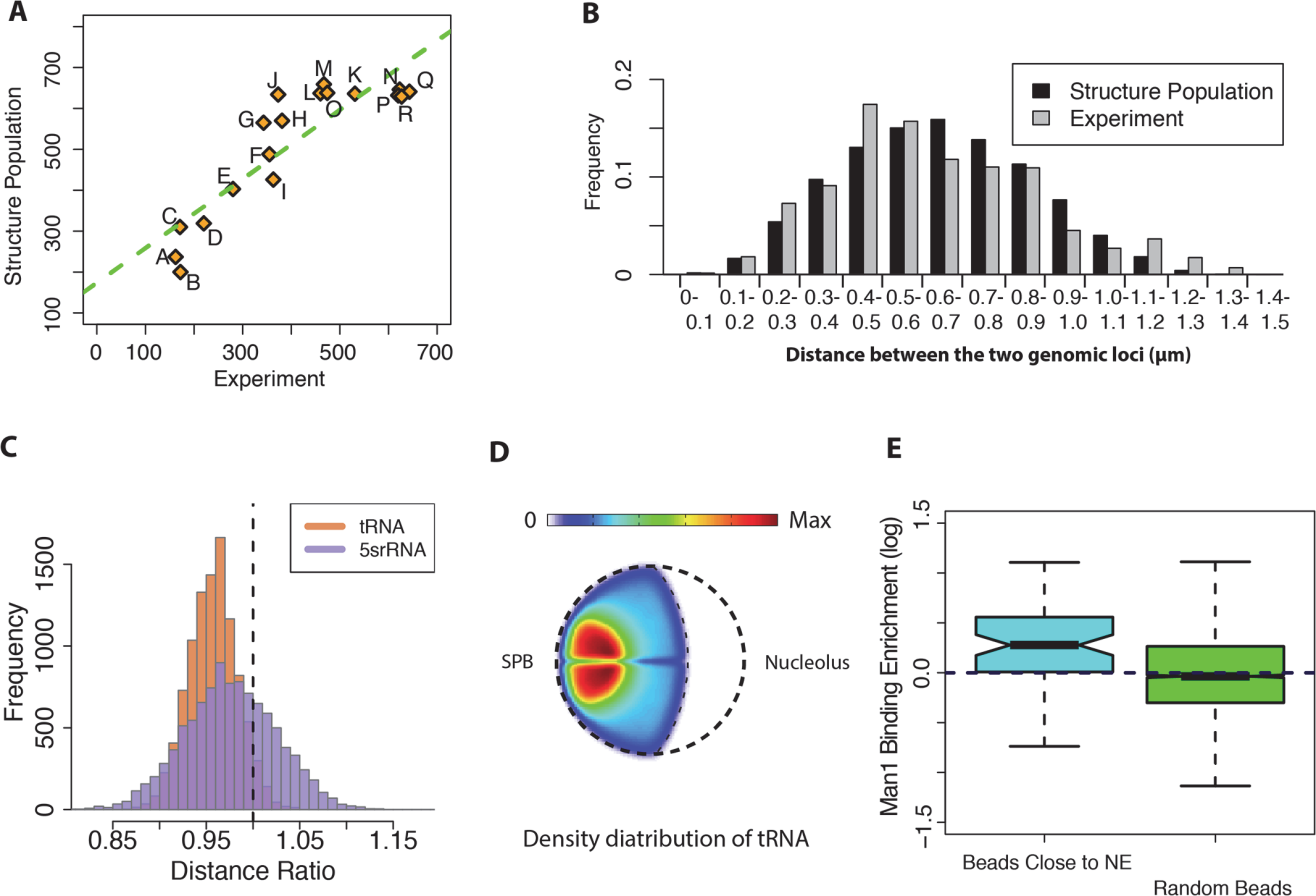
Assessment of the structure population with independent experimental data not included as the input information in the calculations. **(A)** Mean 3D distances for 18 pairs of loci calculated from structure population and determined by independent 3D FISH experiments (R^2^ = 0.77)[23]. The mean distance between two loci is measured as the average distance between the two corresponding chromosome beads in all structures of the population. **(B)** Histogram of the distance distribution between locus chr2 (3094994bp to 3116383bp) and locus chr3 (1404306bp to 1441994bp) in the structure population and FISH experiments [23]. The correlation between the two histograms is calculated as the correlation of the pairwise frequency values between experiment and structure population, *r*_*C*_ = 0.93 (p-value<1E-8). **(C)** Spatial clustering of Pol-III transcribed genes (p-value < 1E-16 for both tRNA and 5srRNA). The histograms show the distribution of the mean pair distance ratio between a set of specific sites (e.g. Pol-III transcribed sites tRNA sites or 5sRNA sites) and all sites in the structures of the population. The distance ratio histograms are generated as follows: For a given structure in the population the mean pair distance between a set of specific loci (e.g. all early replication origins) is calculated. This distance is divided by the mean pair distance of all sites in the same structure to get a distance ratio. The distribution of the distance ratio is then obtained from all structures in the population. The vertical line represents the expected distance ratio if genes are randomly distributed. **(D)** Combined localization probability density (LPD) plot for the 2D distribution of all tRNA sites in fission yeast from our structure population (S1 Text). The density is represented by the color ranging from blue to red. The plot shows that tRNA genes have the highest density close to SPB region. **(E)** Enrichment for chromatin—Man1 protein binding. (Left panel) Enrichment of Man1-binding signal from DamID experiments in the 100 beads that show the shortest average distance to the NE in the structure population. (Right panel) Man1-binding enrichment of randomly selected domains. The results show significantly higher Man1 enrichment in beads closest to NE compared to randomly selected beads (p-value<1E-6, Cohen’d = 0.66).

After establishing the agreement of chromatin contact frequencies in model and experiment we now focus on 3D genome structural features. In independent 3D FISH experiments [23] the average distances of 18 gene pairs were measured [23]. The average loci distances in the structure population were in excellent agreement with experiment (R^2^ = 0.77, Fig. 2A), even though no information about the FISH distances was used when generating the models. Also the distribution of the distances in the structure population agreed well with those from a set of FISH experiments (Fig. 2B) (Pearson correlation of 0.93), indicating that the random encounter of constrained chromosomes can reproduce the data.

### Nuclear localization of PolIII transcribed genes (tRNA, 5sRNA)

FISH experiments showed Pol III-transcribed genes (such as tRNA and 5srRNA) to be spatially clustered, preferentially at centromeric regions [41–43]. Therefore, we calculated the average pairwise 3D distances between all tRNA genes in the structure population and compared the resulting distance distributions with those from randomly selected genome sites. The average 3D distances between tRNA genes were significantly smaller than randomly selected loci (Fig. 2C,D). Similarly, also 5srRNA were spatially clustered in the structure population (Fig. 2C and S2A Fig.). To eliminate the bias of having sites clustered in genomic sequence, we also calculated the average distances between only those Pol III-transcribed gene pairs that were located on different chromosomes. Even for this reduced set we still obtained significant 3D spatial clustering (S2B Fig.). When comparing tRNA clustering between structure populations generated with different constraint types, it becomes evident that tRNA clustering is mainly driven by centromere constraints (‘C Model’ in S2B Fig.). Pol III-transcribed genes were significantly closer to the SPB compared to randomly selected sites (S2C Fig.). Our results indicate that geometric constraints alone (i.e. in particular centromere clustering around the SPB) will increase the probability for Pol III-transcribed genes to be in spatial proximity to each other in 3D space, even if these genes are located on different chromosomes.

### Gene loci-NE distances

DamID experiments reveal the probability of a locus to be close to the NE by measuring its binding propensity to the lamina-like NE protein Man1 [44]. To test if the loci-NE distances in our models agree with DamID experiments, we calculated the average distance of each locus to the NE. The 10% loci with the shortest NE distances in our structure population were selected as loci with the highest likelihood to be positioned close to the NE. We then calculated the enrichment of Man1 binding loci in this set and compared it to a set of randomly selected loci. The set of loci detected to be closest to the NE had a significantly higher MAN1 binding signal enrichment compared to randomly selected sets of loci (p-value < 1E-4) (Fig. 2E).

### Genome structure comparison between fission and budding yeast

We showed that a structure population with constrained but otherwise random chromosome chains combined with the natural gene positioning on chromosomes reproduced many known features of the fission yeast genome organization. We previously showed a similar result for budding yeast[29]. The chromosome organizations between the two yeasts are largely different. We now compare the structure populations of the fission yeast genome with the one previously generated for the budding yeast [29].

### Chromosome Territories

**Fig 3:**
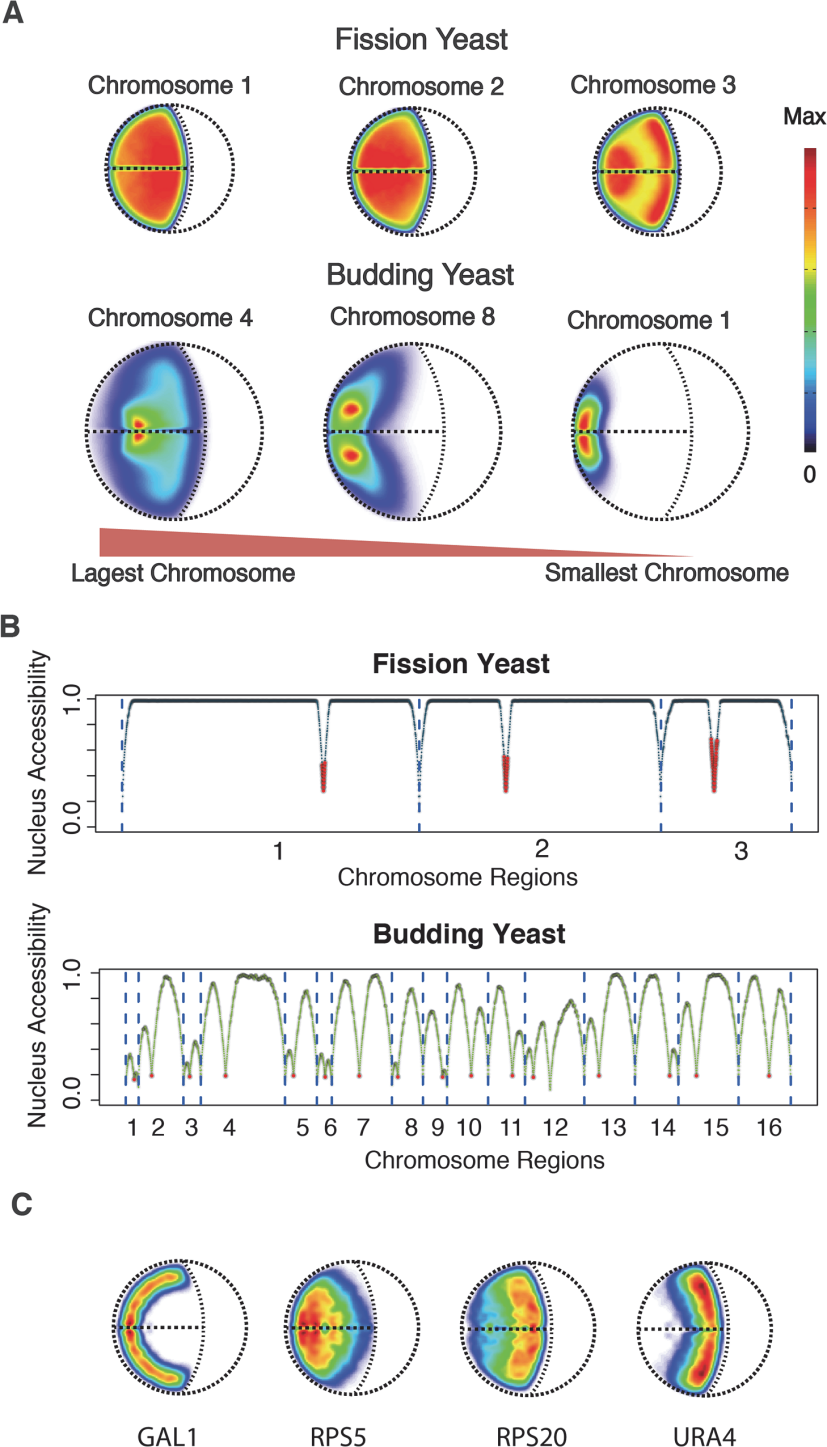
Chromosome and gene territories analysis of fission yeast and budding yeast. **(A)** Chromosome localization probability density (LPD) plots for fission yeast (top panel) and selected chromosomes in budding yeast for comparison (lower panel)[29]. The chromosomes are ordered by their size from largest (left) to smallest chromosomes (right). (**B**) Comparison of the nucleus accessibility of genomic regions between fission yeast and budding yeast (S1 Text). The higher the accessibility, the more space it can explore the nucleus. The red dots in red represent the centromeric locations. (**C**) Gene localization probability density (LPD) plots for four genes in fission yeast. Their orthologous genes were also analyzed in the budding yeast genome models [29].

Chromosome territories were analyzed by calculating the combined location probability density (LPD) of all regions in a chromosome (S1 Text). In fission yeast, the LPD of the large chromosomes 1 and 2 are almost uniformly distributed over the entire nucleus with only slight increase of the LPD closer to the SPB (for chromosome 2), and a slight increase of LPD at the nucleolus for chromosome 1 (Fig. 3A). Only chromosome 3 showed a larger variance in LPD with some increased values at the SPB and the nucleolus, due its smaller size and constraining of the rDNA genes to the nucleolus (Fig. 3A). Moreover, almost all chromatin regions of all three chromosomes can freely access the entire nuclear volume (Fig. 3B top panel). For instance, ~92% of all chromatin regions in the fission yeast genome can access at least 80% of the nuclear volume (Fig. 3B, S1 Text). In budding yeast all the chromosome territories showed substantially larger LPD variations with distinct maxima, at different nuclear locations (Fig. 3A) [29]. Only a relatively small fraction (~32%) of all chromosome regions can access at least 80% of the nuclear volume (Fig. 3B bottom panel). These large differences are mainly due to the substantially smaller but also more variable length of the chromosome arms in budding yeast.

We then analyzed how much a chromosome’s LPD varies if it is calculated from a structure population with or without the remaining chromosomes in the nucleus. In fission yeast the location and extension of a chromosome’s LPD is not affected by the presence of all the other chromosomes (S3 Fig.). In contrast, in budding yeast a chromosome’s LPD dramatically changes with the presence of all other chromosomes in the nucleus [29].

### Gene Territories

To analyze the spatial localization of individual genes we determined the nuclear territories of four genes, for which nuclear territories of their orthologous genes have been previously determined in budding yeast [12,29]. The LPD of these genes reveal preferred locations in the fission yeast nucleus as seen by the LPD maxima of these genes (Fig. 3C). However, the gene territories are significantly more diffuse in fission than in budding yeast. For instance, the gene RPS20 can generally access almost 99% of the nuclear volume while the orthologous gene in budding yeast is substantially more restricted and can access only 29% of the nuclear volume (Table 3). Also, the actual gene locations can be quite different in the two yeast species. For instance, the most dramatic difference among the four genes is seen for the gene RPS20, which is located towards the nucleolus in fission yeast and shows a very large gene territory (Fig. 3C). However, in budding yeast the gene territory of the orthologous gene is quite focused and located close to the spindle pole body[29].

**Table 3:** Nuclear accessibility for orthologous genes in the fission yeast and the budding yeast.

	*GAL1*	*RPS5*	*RPS20*	*URA4/URA3*
*Fission Yeast*	*84%*	*92%*	*64%*	*99%*
*Budding Yeast*	*35%*	*67%*	*42%*	*29%*

The orthologous of URA3 in budding yeast is URA4 in fission yeast.

### Interaction specificity

**Fig 4:**
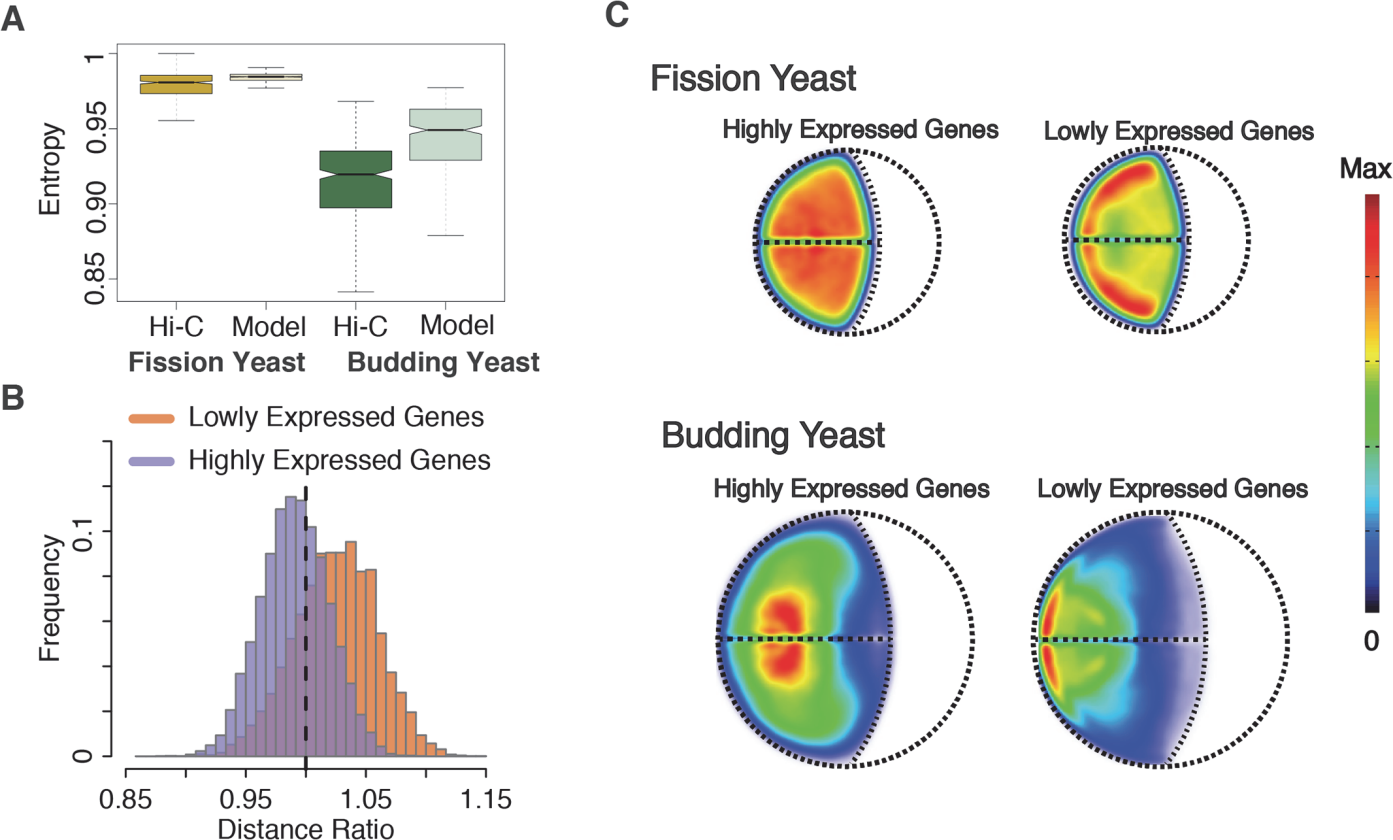
Interaction specificity and gene localization comparison between fission yeast and budding yeast. **(A)** Chromatin interaction specificity analysis. The interaction specificity of a locus is estimated by defining an entropy value, which measures the interaction preference of genomic region with others. The larger the entropy value is, the less specific are its interactions with other genomic regions (S1 Text). Loci in fission yeast show significantly larger entropy values and therefore form more random interactions than in budding yeast, in both Hi-C experiments and structure population (both p-value<1E-16). (**B**) The distribution of the distance ratio for lowly expressed genes and highly expressed genes. Lowly expressed genes are significantly dispersed than randomly selected loci (dashed vertical line), with a p-value<1E-16 and Cohen’s d is 0.72. The highly expressed genes are significantly clustered with a p-value<1E-16 and Cohen’s d is 0.33. (**C**) Combined gene localization probability density (LPD) plots for highly expressed genes and lowly expressed genes in the two yeasts.

We also analyzed the interaction specificity of chromatin regions by defining an ‘interaction entropy’ value for each locus (S1 Text), which measures the preference of a locus to interact with specific loci at increased frequencies. If a locus forms specific interactions its interaction entropy will be low, whereas it will be high if a locus forms interactions to many other loci at similar contact frequencies. Both, in Hi-C experiment and structure population, fission yeast showed significantly larger entropy values (p-value<1E-6) than budding yeast (Fig. 4A). This result confirms that fission yeast chromatin interactions show lower interaction specificity, and are substantially more variable than those in the budding yeast, which indicates that the fission yeast genome organization is less structured than that of budding yeast[29].

### Genome structure and gene expression

In mammalian cells transcriptionally active genes can be co-localized to nuclear sites referred to as transcription factories [4,45,46]. Data from Hi-C experiments indicated a co-location of co-transcribed genes also in fission yeast [23]. We now investigate whether genomic regions that contain highly expressed genes have a tendency to be co-localized also in our structure populations, even though no constraints were imposed between them. We defined two sets of genes, one containing the top 100 ranked genes based on their expression levels in G1 phase and one set containing the bottom 100 ranked genes [23,47]. For both fission and budding yeast, the average 3D distances between highly expressed genes in the structure populations are significantly smaller than those of the lowest expressed genes (Fig. 4B). When plotting the combined LPD for all the genes in each set it is evident that for both yeast types highly expressed genes are localized towards the nuclear interior, while lowly expressed genes reside towards the outer regions close to the NE and SPB (Fig. 4C, S4A Fig.). This finding confirms experimental observations about the preferred location of highly and lowly expressed genes[44], and demonstrates that differences in the nuclear locations between highly and lowly expressed gene sets must be pre-disposed by their sequence positions along the chromosome arms. Indeed, when comparing the distribution of sequence distances of the two gene sets to their respective centromeres and telomeres it becomes evident that the highly expressed genes have a significantly lower genomic distance to centromeres, which resulted in clustering of these genes in 3D space when centromere constraints are imposed (p-value = 0.05, S4B Fig.). The lowly expressed genes have significantly smaller genomic distances to the telomeres, compared to randomly selected loci, which results in a location preferentially close to NE when telomere-NE constrains are imposed (p-value = 0.003, S4C Fig.).

### Nuclear localizations of functionally related genes

**Fig 5:**
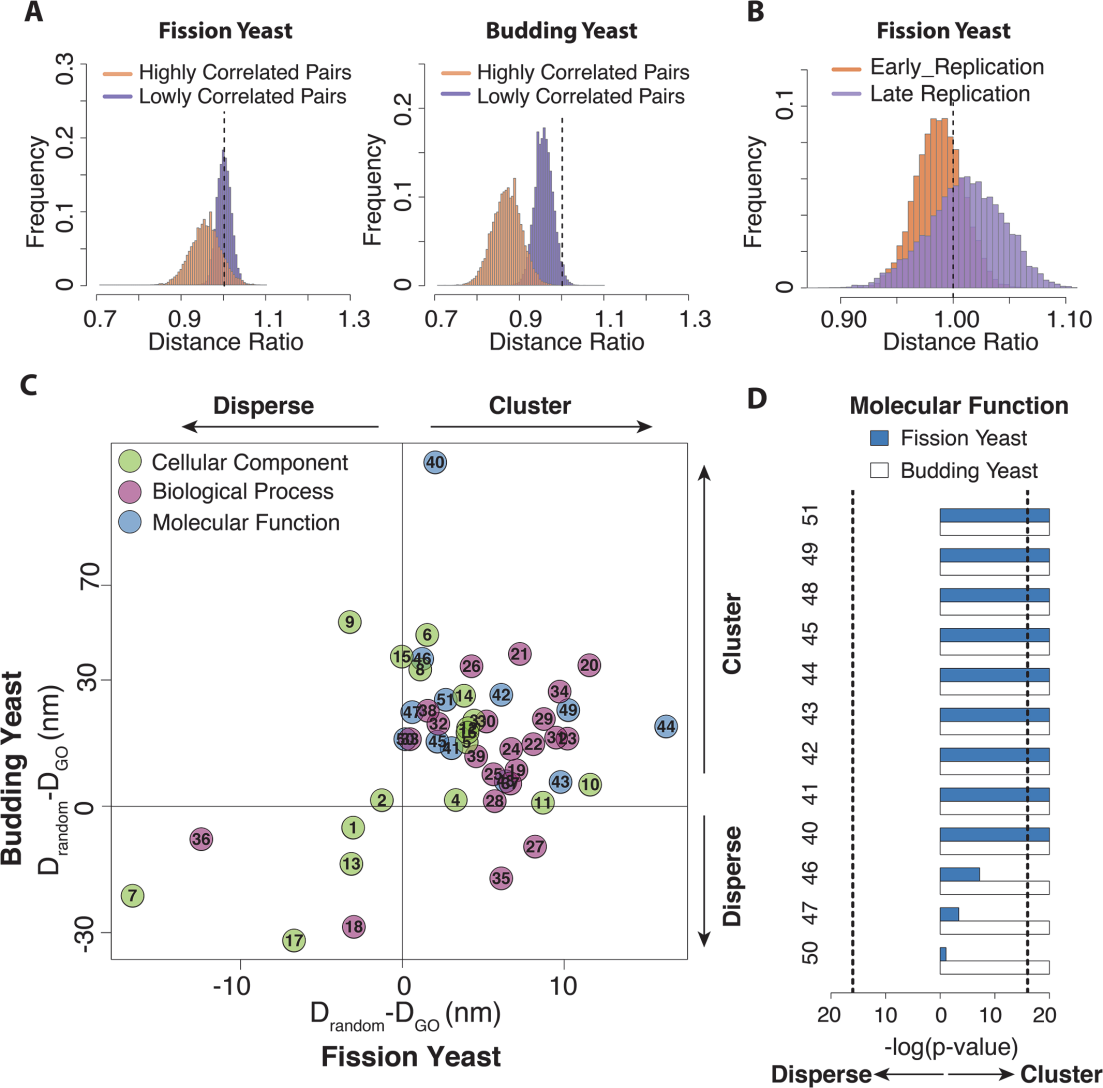
Functional related genes tend to be spatially clustered in both fission yeast and budding yeast. **(A)** Spatial clustering of functional related genes in fission and budding yeast. The histogram shows the distribution of the mean pair distance ratio between a set of functionally related genes, as defined by genetic interaction experiments (S1 Text) and all the sites in the structures population. The histograms are generated as described in Fig. 2D. Genes with low functional correlation score are less clustered compared to related genes with high functional correlation. The Cohen’d for highly functional correlated gene pairs is 1.34 and 3.85 for the fission yeast and the budding yeast (p-value<1E-16 for both cases). The Cohen’d for lowly functional correlated genes is 0.13 and 2.17 for the fission yeast and the budding yeast, respectively (p-value<1E-16 for both cases). **(B)** Histograms of the distributions of the mean pair distance ratio between the set of early replication origins and all the sites in the structure population. A corresponding histogram is shown also for late replication origins. Early replication origins are spatially clustered in fission yeast (p-value<1E-16, Cohen’d = 0.59), while late replication site show statistically significant larger average distances than randomly selected sites (p-value<1E-16, Cohen’d = 0.4) **(C)** Comparison of the clustering of genes in the same GO categories between fission yeast and budding yeast. The test is based on 51 GO categories, which contain sufficient amount of genes in both yeast types. Plotted is the difference D_GO_—D_random_ between the average pairwise 3D distances of the genes in a GO category (D_GO_) and the average pairwise 3D distances between randomly selected gene sites (D_random_). If the difference D_random_-D_GO_ is larger than 0, genes in the GO category is defined as clustered (p-value<-1E16), and If the difference is smaller than 0, genes in the GO category are considered to be”dispersed” (p-value<-1E16). Each point representing a GO category is colored by their functional categories, such as cellular component, biological process and molecular function. **(D)** The clustering of functional categories is highly significant. Shown is a selection of GO categories under the term molecular functions. The dashed line indicates a p-value of 1E-16. The—log(p-value) is trimmed at maximally p-value = 1E-20. The numbers on the left of the figure represent GO categories as labeled in S1 Table. For all the genes in the class “Molecular Function”, 10 of 10 GO categories are significantly clustered in budding yeast, while genes in 7 out of the 10 same GO categories are significantly clustered in fission yeast.

In budding yeast, functionally related genes tend to be co-localized [6,26]. We next study, if such gene co-localization can also be reproduced in our structure populations of fission and budding yeast even though no constraints were imposed between functionally related genes. Genetic interaction (GI) experiments provided a large number of gene pairs with related functions [48–50]. Based on these experiments, we selected 2 sets of gene pairs, one with functional correlated and one with functional uncorrelated gene pairs (S1 Text). Interestingly, for both, fission and budding yeast, functionally related genes have shorter averaged 3D distances in the structure population than functionally unrelated genes (Fig. 5A). This observation indicates that random chromosome conformations subject to a few geometric constrains may in part explain these structure-function correlations.

Moreover, we also observed that early replication origins in fission yeast are spatially clustered in our structure population. The average pairwise 3D distances of early replication origins are significantly smaller compared to a set of randomly selected sites (Fig. 5B) [51]. In contrast, the averaged pairwise 3D distances of late replication sites are significantly larger compared to randomly selected loci, indicating that late replication origins are more dispersed in 3D space. We previously found the same behavior in budding yeast, even though the two yeast types have different locations of their replication origins and very different chromosome organizations[29].

### Association among genes in same ontology groups

Finally, we analyzed the co-locations of genes classified in the same gene ontology group (GO) (http://www.pombase.org and http://www.yeastgenome.org/). To have sufficient sampling for our analysis, we selected GO classes containing at least 50 and up to 500 genes, which resulted in the selection of 51 GO terms for each yeast species (S1 Table). For each set of genes in a GO category, we calculated the average pairwise 3D distance in the structure population. We then compared these data with those of randomly selected set of loci (Fig. 5C). If a gene set had a significantly smaller average 3D distance than a set of randomly selected loci (based on a p-value<1E-16), we considered these genes to be “clustered”. Genes were considered to be “dispersed” if their average distance was significantly larger than randomly selected loci (based on a p-value<1E-16). Interestingly, genes in the same GO category tend to be clustered for both yeast types. In fission yeast 36 out of 51 GO categories show significant gene clustering, while 41 out of the 51 GO categories show significant gene clustering in the budding yeasts (Table 4). This observation agrees with our finding that functional related genes are more clustered in 3D space. Our structure population also reveals a similarity in the clustering property for the same GO categories in both yeast species. Among the 51 GO categories, 38 GO categories (>74%), showed identical gene clustering behavior in both yeast species (32 clustered and 6 dispersed GO categories) (Fig. 5D, S5 Fig., Table 4). Therefore, the clustering properties must be pre-disposed in the sequence position of these genes. Indeed, when calculating the average sequence distances of genes, it is evident that genes in most GO categories are already clustered at sequence level for both yeast species (S2 Table).

**Table 4:** Clustering properties of genes in 51 GO categories.

	*Fission Yeast*			
*Budding Yeast*		*Clustered*	*Random*	*Dispersed*
	*Clustered*	32	*7*	*2*
	*Random*	*2*	*0*	*0*
	*Dispersed*	*2*	*0*	*6*

## Conclusions

Here, we studied the genome organization of fission yeast and characterized the chromatin contact patterns, and nuclear territories of chromosomes and gene loci, which emerge when chromosomes are allowed to behave as constrained but otherwise randomly configured flexible polymer chains. This model is sufficient to explain in a statistical manner many experimentally determined distinctive features of the fission yeast genome organization, such as Hi-C contact patterns, including the enhanced interaction frequencies between telomeres; and the co-location of some co-expressed genes and co-locations of functionally related genes, including early replication start sites, tRNA genes, and 5sRNA genes. Our findings demonstrate that some structure-function correlations can be explained as a consequence of random chromatin collisions driven by a few geometric constraints combined with the natural gene positioning on the chromosomes. Distinguishing such “driver” interactions from “passenger” interactions is key in understanding the principles of spatial genome organization and genome structure-function correlations.

We also performed a comparative genome structure analysis between fission and budding yeast, for which similar organization principles have been described previously[29]. Despite similar organizing principles, large differences exist between fission and budding yeast genome structures. In fission yeast large fractions of the chromosomes can almost freely access the entire nuclear volume and gene territories are diffuse. In contrast, the budding yeast genome is substantially more structured with more focused gene territories and most chromosome regions can only access a restricted region of the nucleus. Moreover, in budding yeast the inter-chromosomal interaction patterns are highly structured leading to cross-shaped patterns in the contact map. These cross-shaped interaction patterns are due to an exclusion volume effect when centromeres of all the 16 chromosomes compete for the limited space around the SPB[29]. Because in fission yeast only 3 chromosomes cluster at the SPB, such interaction patterns are not observed despite imposing identical geometric constraints. Moreover, due to the substantially longer chromosome arms combined with a smaller nuclear radius, locations of gene loci are more disperse across the nucleus. Therefore, in fission yeast specific inter-chromosomal interactions are mainly restricted to regions directly adjacent to centromeric and telomeric regions.

Despite the differences in genome organization, many functional similarities prevail. For instance, in both yeast types the average 3D distances between highly expressed genes are significantly smaller than those of the lowest expressed genes. The observed co-localization between functionally related genes in fission yeast is mainly due to clustered gene locations along the chromosome sequence and the fact that they are enriched towards either the centromeres or telomeres.

Finally, our work also highlights that experimental data on fission yeast is consistent with a population of genome structures that can significantly vary between them. Such an observation cautions against using structure models based on ensemble-averaged experimental data. Such models may not be able to capture accurately many of the structural properties of the genome.

## Supporting Information

S1 Fig Genomic distance vs. contact frequency plot for the fission yeast and budding yeast. The contact frequency value is calculated the average contact frequency for all pair of genomic regions for a given genomic distance. The fitted line is generated using support vector regression with radial kernel. Experimental data shows a high interaction frequency for two ends of chromosomes, which represent the contact frequency between two telomeres. We could also observe the same effect in our CNR model but not in Control model.
(TIF)

S2 Fig Pol-III transcribed genes localization property. **(A)** Density plot of 5srRNA in the nucleus in 2D. The more red the color is, the high possibility that 5srRNA would occur. **(B)** The clustering analysis for Pol III genes considering only inter-chromosomal pairwise distance for different models. The star(*) symbol represents our targeted regions showing a significant difference (p-value<1E-16) in clustering property from randomly select regions. For both tRNA and 5srRNA, it shows significant clustering property for all models except for Control model. However, Cohen’s d calculation shows that the effect size of the clustering property of different constraints contribute differently. For tRNA genes, we can see that they show an effective clustering property in C, CNR model (Cohen’s d > 0.4). For 5srRNA we can see that it shows it shows effective clustering property only in CNR model. **(C)** Histogram of distance of Pol III selected genomic regions, tRNA and 5srRNA, to the SPB normalized by the randomly selected genomic regions (both p-value<1E-16). The Cohen’s d analysis shows a strong clustering property for both genes, Cohen’s d for tRNA = 2.42 and 5srRNA = 0.62.
(TIF)

S3 Fig LDP plot for chromosomes from our single chromosome structure populations. Each chromosome is subject to all geometric constraints, but without the presence of other chromosomes in the nucleus.
(TIF)

S4 Fig Gene expression and location preference (A) Average distances between genes and the NE for lowly expressed genes and highly expressed genes, respectively, in fission and budding yeast. For both yeasts, lowly expressed genes are significantly closer to NE than highly expressed genes (both p-value<1E-16, Cohen’d is 1.73 for the fission yeast and 3.93 for the budding yeast). **(B,C)** The comparison of genomic distances to centromere/telomere between highly/lowly expressed genes and randomly selected loci. **(B)** Highly expressed genes are significantly close to centromeres comparing to randomly selected loci in fission yeast (p-value< = 0.05). There is no significant difference between highly expressed genes and randomly selected loci in terms of distance to telomeres. **(C)** There is no significant different between lowly expressed genes and randomly selected loci in distance to centromeres. Lowly expressed genes are significantly close to telomeres comparing to randomly selected loci (p-value< = 0.05).
(TIF)

S5 Fig The significance of clustering of GO categories under the category of cellular component and biological process. The dash line represents p-value equals to 1E-16. Here the—log(p-value) is trimmed at maximally p-value = 1E-20. For GO categories in cellular component, genes in 7 GO categories show clustering property in both yeasts, while genes in 4 GO categories show dispersed property in both yeasts. For GO categories in biological process, genes in 16 GO categories show clustering property in both yeasts, while genes in 2 GO categories show dispersed property in both yeasts.
(TIF)

S1 Text Supplementary Material. (PDF)

S1 Table GO categories information for structure comparison. (PDF)

S2 Table Pairwise genomic distance (intra-chromosomal) of genes in GO categories in the fission yeast and the budding yeast. The genomic distance is compared with those obtained from the random select loci.
(PDF)
