# Supplementary figures and images for: Comparative 3D Genome Structure Analysis of the Fission and the Budding Yeast
Source: PLoS One. 2015 Mar 23;10(3):e0119672. doi: 10.1371/journal.pone.0119672 (PMC4370715; doi:10.1371/journal.pone.0119672)

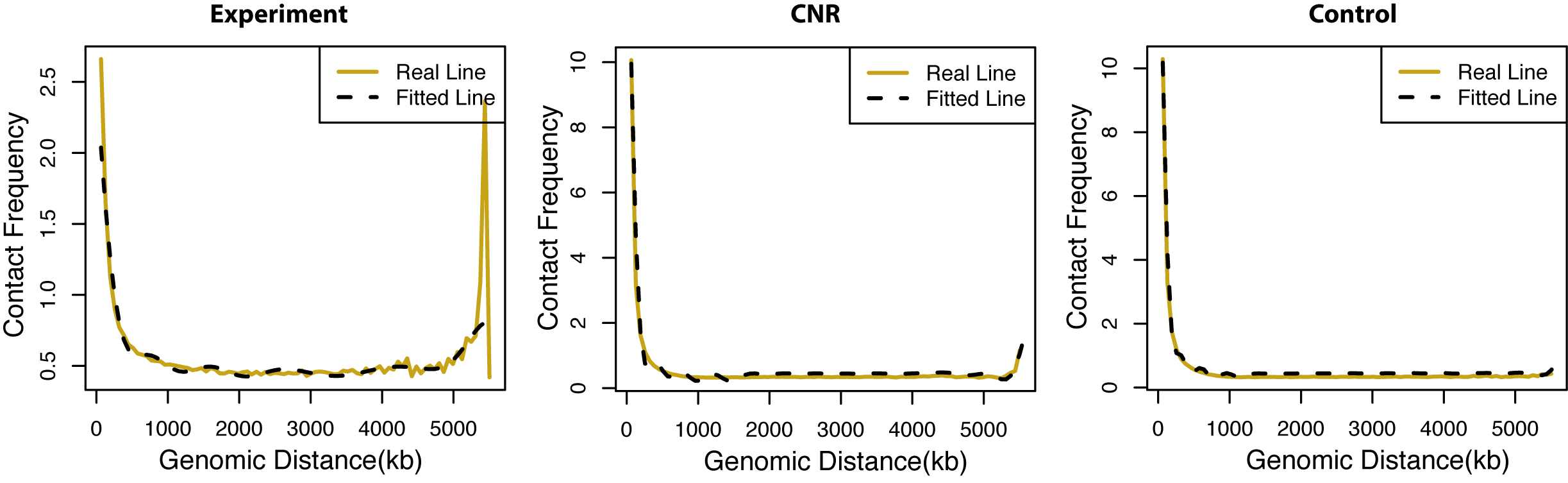

Supplement: S1 Fig — The contact frequency value is calculated the average contact frequency for all pair of genomic regions for a given genomic distance. The fitted line is generated using support vector regression with radial kernel. Experimental data shows a high interaction frequency for two ends of chromosomes, which represent the contact frequency between two telomeres. We could also observe the same effect in our CNR model but not in Control model. (TIF) [file pone.0119672.s001.tif]

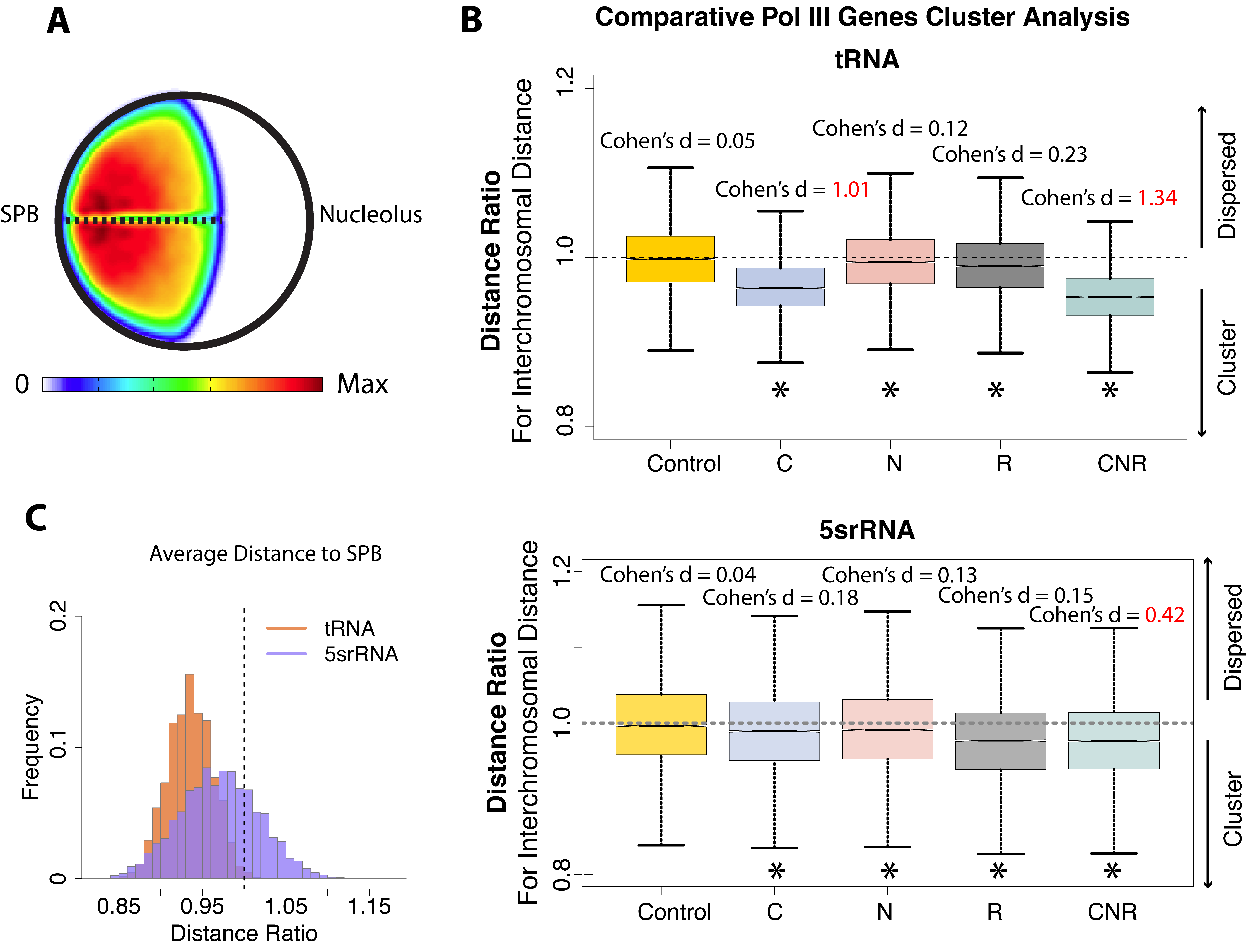

Supplement: S2 Fig — (A) Density plot of 5srRNA in the nucleus in 2D. The more red the color is, the high possibility that 5srRNA would occur. (B) The clustering analysis for Pol III genes considering only inter-chromosomal pairwise distance for different models. The star(*) symbol represents our targeted regions showing a significant difference (p-value<1E-16) in clustering property from randomly select regions. For both tRNA and 5srRNA, it shows significant clustering property for all models except for Control model. However, Cohen’s d calculation shows that the effect size of the clustering property of different constraints contribute differently. For tRNA genes, we can see that they show an effective clustering property in C, CNR model (Cohen’s d > 0.4). For 5srRNA we can see that it shows it shows effective clustering property only in CNR model. (C) Histogram of distance of Pol III selected genomic regions, tRNA and 5srRNA, to the SPB normalized by the randomly selected genomic regions (both p-value<1E-16). The Cohen’s d analysis shows a strong clustering property for both genes, Cohen’s d for tRNA = 2.42 and 5srRNA = 0.62. (TIF) [file pone.0119672.s002.tif]

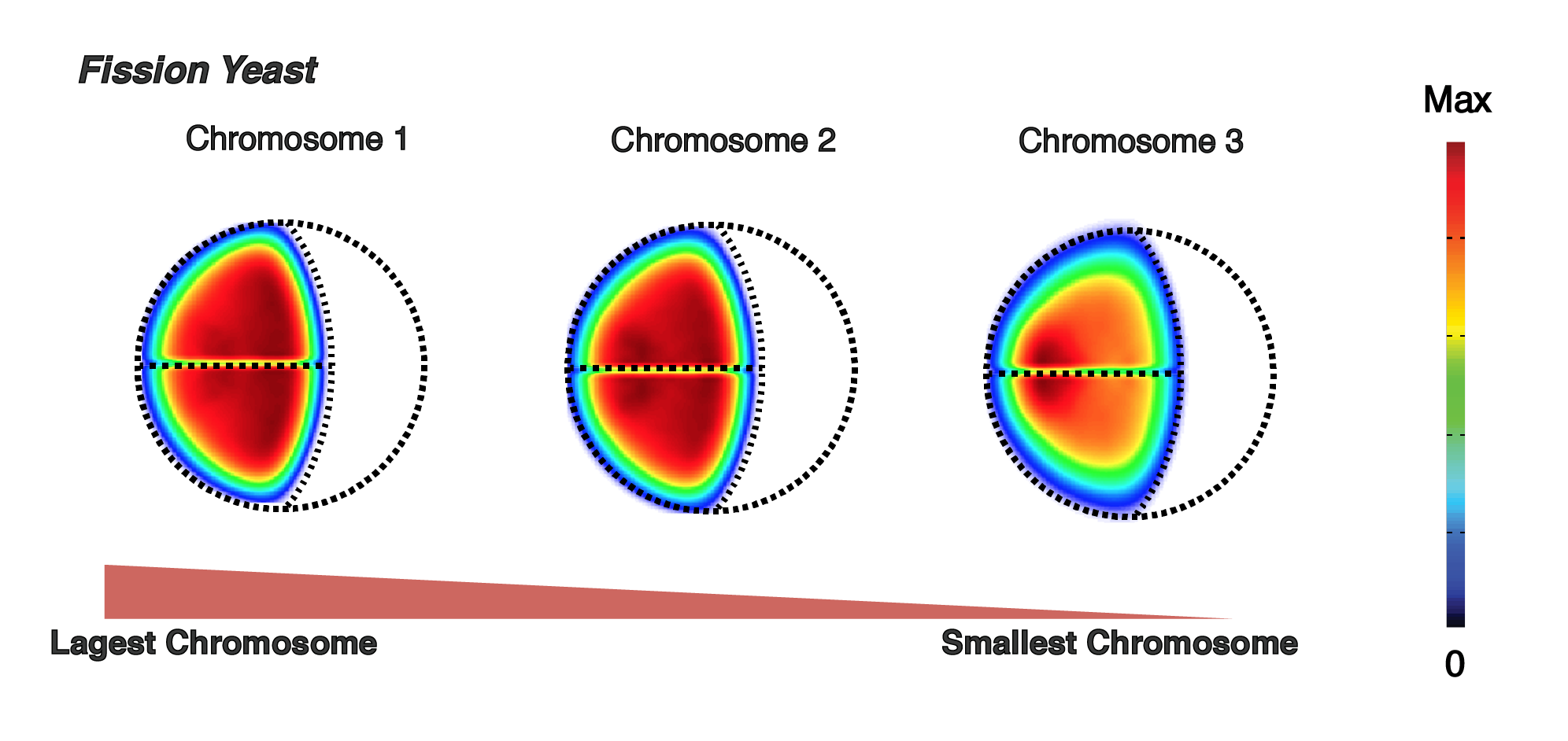

Supplement: S3 Fig — Each chromosome is subject to all geometric constraints, but without the presence of other chromosomes in the nucleus. (TIF) [file pone.0119672.s003.tif]

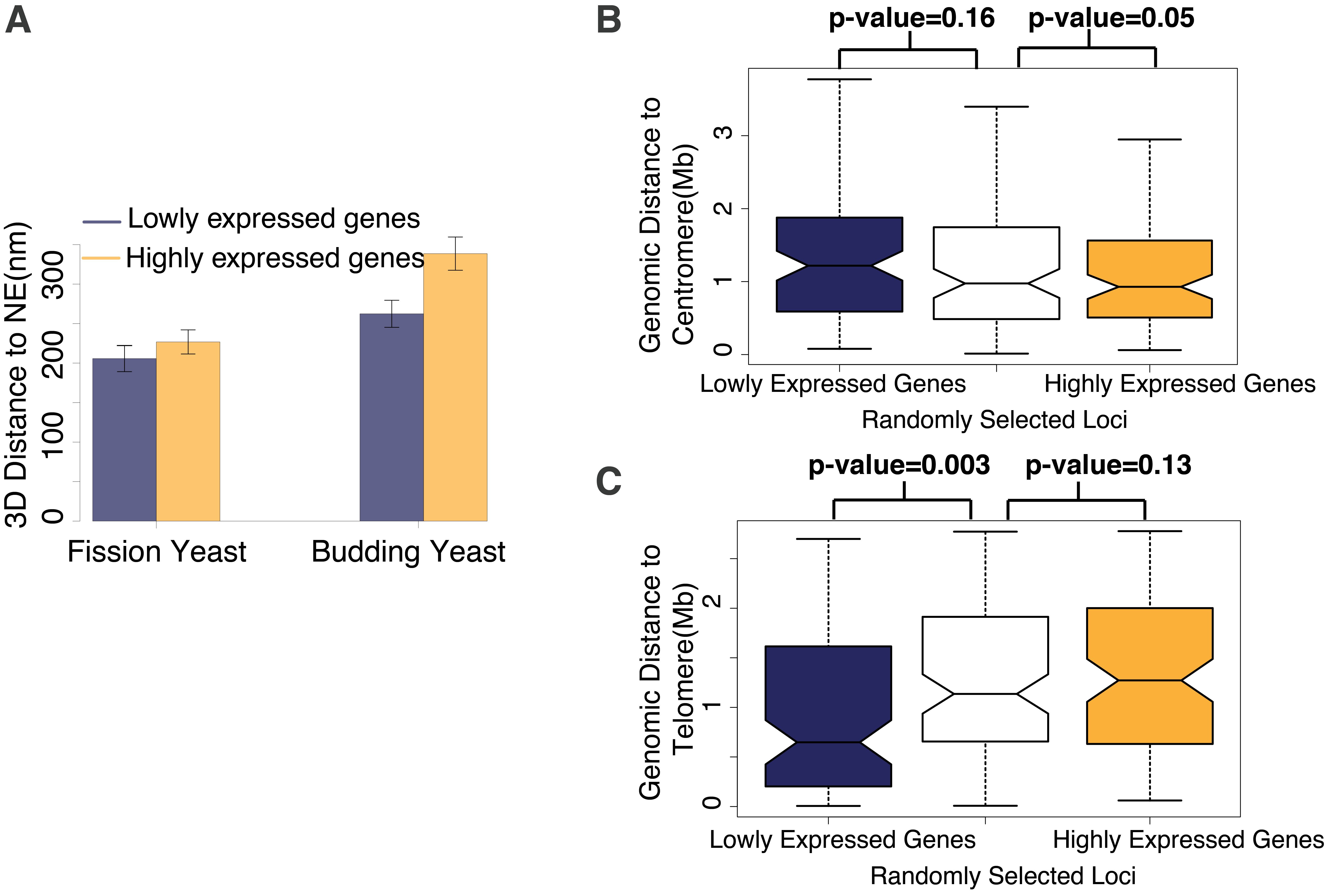

Supplement: S4 Fig — For both yeasts, lowly expressed genes are significantly closer to NE than highly expressed genes (both p-value<1E-16, Cohen’d is 1.73 for the fission yeast and 3.93 for the budding yeast). (B,C) The comparison of genomic distances to centromere/telomere between highly/lowly expressed genes and randomly selected loci. (B) Highly expressed genes are significantly close to centromeres comparing to randomly selected loci in fission yeast (p-value< = 0.05). There is no significant difference between highly expressed genes and randomly selected loci in terms of distance to telomeres. (C) There is no significant different between lowly expressed genes and randomly selected loci in distance to centromeres. Lowly expressed genes are significantly close to telomeres comparing to randomly selected loci (p-value< = 0.05). (TIF) [file pone.0119672.s004.tif]

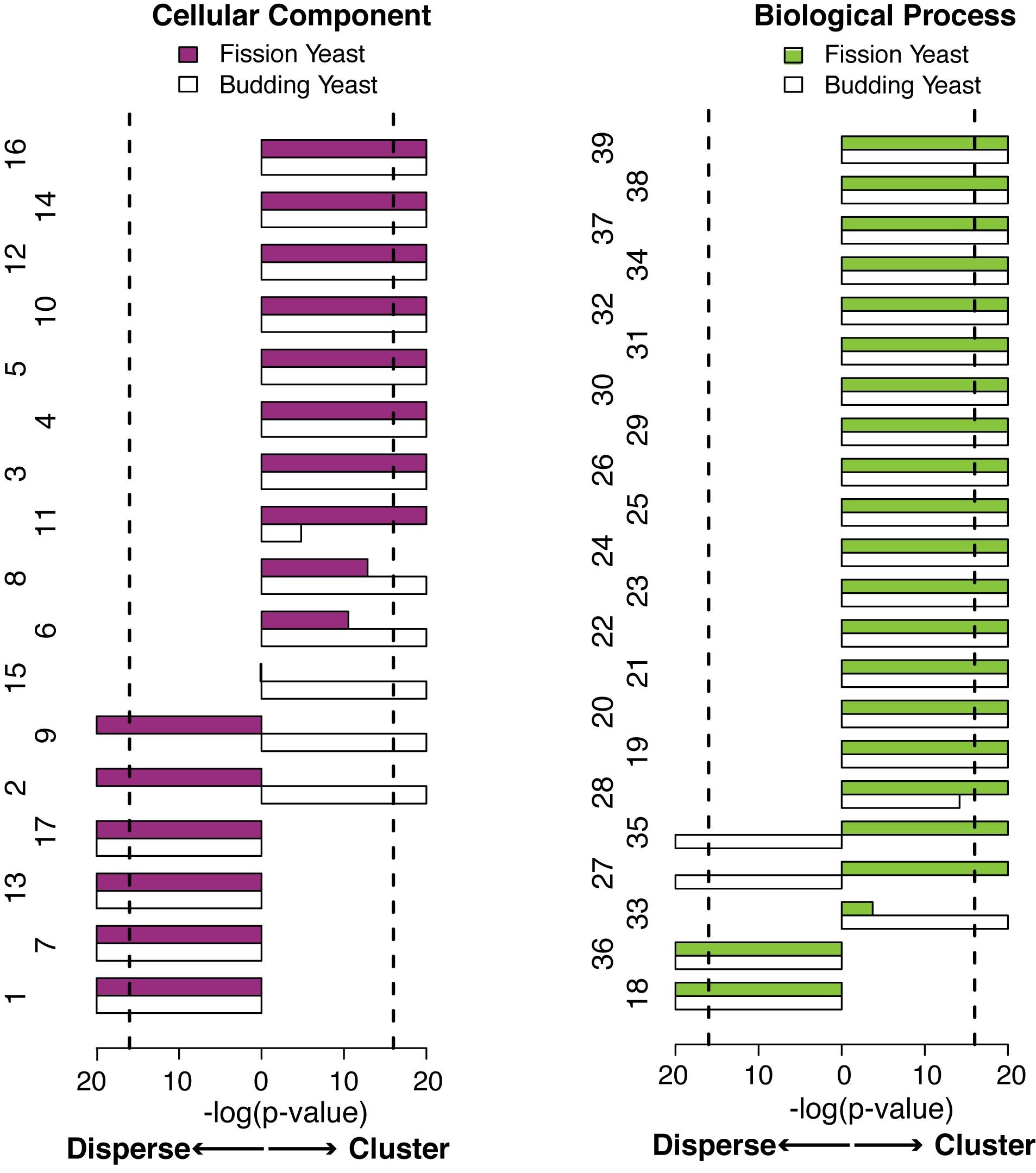

Supplement: S5 Fig — The dash line represents p-value equals to 1E-16. Here the—log(p-value) is trimmed at maximally p-value = 1E-20. For GO categories in cellular component, genes in 7 GO categories show clustering property in both yeasts, while genes in 4 GO categories show dispersed property in both yeasts. For GO categories in biological process, genes in 16 GO categories show clustering property in both yeasts, while genes in 2 GO categories show dispersed property in both yeasts. (TIF) [file pone.0119672.s005.tif]
