# Supplementary material for: Comparative 3D Genome Structure Analysis of the Fission and the Budding Yeast
Source: PLoS One. 2015 Mar 23;10(3):e0119672. doi: 10.1371/journal.pone.0119672 (PMC4370715; doi:10.1371/journal.pone.0119672)
Supplement: S1 Text — (PDF) [file pone.0119672.s006.pdf]

SUPPLEMENTARY MATERIAL

Structure Data

The model structures are accessible at the website
http://web.cmb.usc.edu/people/alber/Data/Gong_etal_pombe_structures.tar.gz

Representation of Genome-wide Contact Frequency Map at 96 kb Resolution

$$\mathbf{C}_{130}^{96\text{kb}} = (c_{ij})_{130\times 130}$$

The contact frequency map at 96kb resolution is defined as a 130×130 matrix. Each cell in the matrix represent the contact frequency between two 64kb of consecutive genomic regions. For our structure population, the contact frequency c_{ij} is equal to the total sum of observed contacts between any one of the beads in one region (i) to any one of the beads of the second region (j). For Hi-C experimental contact frequency map, c_{ij} is defined as the sum of all the physical proximity values between region (i) and region (j).

After we obtain the contact frequency map $\mathbf{C}_{130}^{96\text{kb}}$, a normalization is processed following protocol provided by Imakaev, et al [1]. All the matrix comparison is performed based on normalized contact frequency map.

Matrix Correlation Measurement

Let $\overline{\mathbf{C}}_n^{\text{Exp}} = (\overline{c}_{ij}^{\text{Exp}})_{n\times n}$ and $\overline{\mathbf{C}}_n^{\text{P}} = (\overline{c}_{ij}^{\text{P}})_{n\times n}$ represents the contact frequency matrix for experimental and structure population. The Pearson's correlation coefficient between the two matrices is as follows.

$$r = \frac{n^2\sum_{i=1}^{n}\sum_{j=1}^{n}\overline{c}_{ij}^{\text{P}}\overline{c}_{ij}^{\text{Exp}} - (\sum_{i=1}^{n}\sum_{j=1}^{n}\overline{c}_{ij}^{\text{P}})(\sum_{i=1}^{n}\sum_{j=1}^{n}\overline{c}_{ij}^{\text{Exp}})}{\sqrt{n^2\sum_{i=1}^{n}\sum_{j=1}^{n}(\overline{c}_{ij}^{\text{P}})^2 - (\sum_{i=1}^{n}\sum_{j=1}^{n}\overline{c}_{ij}^{\text{P}})^2}\sqrt{n^2\sum_{i=1}^{n}\sum_{j=1}^{n}(\overline{c}_{ij}^{\text{Exp}})^2 - (\sum_{i=1}^{n}\sum_{j=1}^{n}\overline{c}_{ij}^{\text{Exp}})^2}},$$

Localization Probability Density (LPD) of Gene and Chromosome

To visualize loci localization in the nucleus, we calculate localization probability density (LPD) maps from our structure population. We first collect all the position information (x,y,z) of target beads and projected those 3D coordinates into a 2D space [2]. A density grid projection and normalization is done using the same protocol as the budding yeast analysis[3].

For any given set of genes, we first collect the 3D coordinates (x',y',z') of all genes in all the structure population, and then project them into a 2D space using the following formula:

$$(z, \rho)=(x',\sqrt{y'^2 + z'^2})$$

Next, we perform density grip projection with a grid size as Δ = 10 nm, which results into a 2D grid of 142*142 pixels. Once we map the point (z_c,ρ_c) to a grid, a Gaussian blur is needed using the following formula

$$G_{ij} = \frac{1}{\sqrt{2\pi\sigma^2}} \exp\left(-\frac{(z_c - z_i)^2 + (\rho_c - \rho_j)^2}{2\sigma} \right)$$

where z_c and ρ_c represents the center of the pixel in z and p axis. (*i,j*) denote the neighboring pixels and σ = 30nm.

Normalization is performed after Gaussian blur using following formula

$$\bar{G}_{ij} = \frac{G_{ij}}{\pi\Delta\left(\left(\rho_j + \Delta\right)^2 - \rho_j^2\right)}$$

and Δ is the grid size. Finally, we divide all $\bar{G}_{ij}$ by the maximum of $\bar{G}_{ij}$ so that the maximum value is 1.

Nucleus Accessibility

To estimate how much a locus can explore inside a nucleus we define its nucleus accessibility. For given genomic regions, we first get the 2D space it can explore (the total number of grids with the density value > 0.0001) through LPD analysis. The nucleus accessibility can be calculated as the fraction of total available nuclear space, excluding the nucleolus regions, that is accessible to the regions at 2D space.

Interaction Entropy Calculation

Entropy can be used to measure uncertainty in a random variable, in this case interaction frequency coming from a domain to its partners. The higher the entropy, the more unspecific interactions it has to its partners.

The entropy of bin *i* is defined as $E_i = \frac{\sum_{j=1}^{N_{\text{inter}}} P_{ij} \log_2 \mathrm{P}_{ij}}{\sum_{j=1}^{N_{\text{inter}}} e_{ij} \log_2 e_{ij}}$ where $P_{ij} = \frac{f_{ij}}{\sum_{j}^{N_{\text{inter}}} f_{ij}}$ and the expected uniform distribution values are simply $e_{ij} = 1/N_{\text{inter}}$.

Functional Correlation Gene Pairs from Genetic Interaction Experiment

The functional correlation between two genes is calculated as the cosine correlation or dot product between the two genes' genetic interaction score of all query genes.

$$\mathbf{C}_{i,j} = \frac{\sum_{k=1}^{n} G_{i,k} \bullet G_{j,k}}{\sqrt{\sum_{k=1}^{n} G_{i,k} \bullet G_{i,k}} \sqrt{\sum_{k=1}^{n} G_{i,k} \bullet G_{i,k}}}$$

where *i* and *j* represents the target gene pairs. $G_{i,k}$ represents the genetic interaction score between gene *i* and gene *k*.

The highly correlated gene pairs are selected as the top 300 gene pairs that have the highest correlations (> 0.7 for budding yeast and > 0.55 for fission yeast). The uncorrelated gene pairs are selected for those gene pairs what have correlations ranging from -0.005 to 0.005.

SUPPLEMENTARY REFERENCES

1. Imakaev M, Fudenberg G, McCord RP, Naumova N, Goloborodko A, Lajoie BR, et al. (2012) Iterative correction of Hi-C data reveals hallmarks of chromosome organization. Nat Methods 9: 999-1003.
2. Berger AB, Cabal GG, Fabre E, Duong T, Buc H, Nehrbass U, et al. (2008) High-resolution statistical mapping reveals gene territories in live yeast. Nat Methods 5: 1031-1037.

3. Tjong H, Gong K, Chen L, Alber F (2012) Physical tethering and volume exclusion determine higher-order genome organization in budding yeast. Genome Res 22: 1295-1305.
