# Supplementary material for: Comparative 3D Genome Structure Analysis of the Fission and the Budding Yeast
Source: PLoS One. 2015 Mar 23;10(3):e0119672. doi: 10.1371/journal.pone.0119672 (PMC4370715; doi:10.1371/journal.pone.0119672)
Supplement: S1 Table — (PDF) [file pone.0119672.s007.pdf]

No.	Label	Number Of Genes in Fission Yeast	Number Of Genes in Budding Yeast	Function Category
**1**	GO:0005886	136	401	Cellular Component
**2**	GO:0005794	268	102	Cellular Component
**3**	GO:0005730	309	247	Cellular Component
**4**	GO:0000329	84	139	Cellular Component
**5**	GO:0022625	82	82	Cellular Component
**6**	GO:0005643	52	57	Cellular Component
**7**	GO:0005576	53	91	Cellular Component
**8**	GO:0000139	125	94	Cellular Component
**9**	GO:0031965	82	65	Cellular Component
**10**	GO:0022627	58	62	Cellular Component
**11**	GO:0005816	64	54	Cellular Component
**12**	GO:0005759	80	105	Cellular Component
**13**	GO:0005635	94	58	Cellular Component
**14**	GO:0030479	51	59	Cellular Component
**15**	GO:0005789	277	254	Cellular Component
**16**	GO:0005743	104	155	Cellular Component
**17**	GO:0016020	53	107	Cellular Component
**18**	GO:0006508	69	79	Biological Process
**19**	GO:0002181	154	163	Biological Process
**20**	GO:0000122	78	80	Biological Process
**21**	GO:0043161	54	53	Biological Process
**22**	GO:0006468	105	122	Biological Process
**23**	GO:0006338	133	73	Biological Process
**24**	GO:0016567	98	62	Biological Process
**25**	GO:0006364	130	105	Biological Process
**26**	GO:0045944	72	156	Biological Process
**27**	GO:0007126	56	84	Biological Process
**28**	GO:0006888	73	77	Biological Process
**29**	GO:0032543	113	88	Biological Process
**30**	GO:0006281	98	99	Biological Process
**31**	GO:0006457	82	91	Biological Process
**32**	GO:0006897	56	85	Biological Process
**33**	GO:0006355	151	129	Biological Process
**34**	GO:0016192	71	63	Biological Process
**35**	GO:0030437	79	53	Biological Process
**36**	GO:0055085	102	95	Biological Process

**37**	GO:0015031	62	182	Biological Process
**38**	GO:0006357	125	64	Biological Process
**39**	GO:0006886	153	60	Biological Process
**40**	GO:0004842	70	69	Molecular Function
**41**	GO:0003677	225	197	Molecular Function
**42**	GO:0016887	68	71	Molecular Function
**43**	GO:0003735	213	217	Molecular Function
**44**	GO:0051082	55	87	Molecular Function
**45**	GO:0003723	239	232	Molecular Function
**46**	GO:0003924	58	71	Molecular Function
**47**	GO:0000978	85	86	Molecular Function
**48**	GO:0004674	95	101	Molecular Function
**49**	GO:0001077	110	63	Molecular Function
**50**	GO:0008270	286	312	Molecular Function
**51**	GO:0005525	112	98	Molecular Function
