# Supplementary material for: Comparative 3D Genome Structure Analysis of the Fission and the Budding Yeast
Source: PLoS One. 2015 Mar 23;10(3):e0119672. doi: 10.1371/journal.pone.0119672 (PMC4370715; doi:10.1371/journal.pone.0119672)
Supplement: S2 Table — The genomic distance is compared with those obtained from the random select loci. (PDF) [file pone.0119672.s008.pdf]

	Fission Yeast		
Budding Yeast		*Smaller Pairwise Genomic Distance*	*Larger Pairwise Genomic Distance*
	Smaller Pairwise Genomic Distance	*31*	*3*
	Larger Pairwise Genomic Distance	*11*	*5*
